# Maggot Wound Therapy Associated with Wohlfahrtiimonas chitiniclastica Blood Infection

**DOI:** 10.7759/cureus.12471

**Published:** 2021-01-04

**Authors:** Peter Bueide, Jeff Hunt, Dinesh Bande, Dubert M Guerrero

**Affiliations:** 1 Internal Medicine, University of North Dakota School of Medicine and Health Sciences, Fargo, USA; 2 Internal Medicine, Sanford Health, Fargo, USA

**Keywords:** maggot, wound, bacteremia

## Abstract

Maggot therapy is the intentional application of live, disinfected fly larvae to wounds for debridement and wound care. Although some studies suggest possible beneficial applications for wound healing, subsequent infection is a potential risk of treatment. We present a case of a 70-year-old male with chronic left temporal wound from squamous cell carcinoma treated with maggot therapy complicated by bacteremia with *Wohlfahrtiimonas chitiniclastica (W. chitiniclastica)*. This case illustrates the risk for *W. chitiniclastica* infection associated with maggots used in medical wound therapy of chronic wounds.

## Introduction

Maggot therapy was made popular during the first world war following the observation that soldiers presenting with maggots in their wounds had a decreased mortality rate than their counterparts [1]. On certain occasions, it is still being used as an alternative treatment option for chronic wounds today [2]. However, much remains unknown regarding the biochemical and physiologic mechanisms underlying the process and the potential risks associated with it. Here, we describe a case of *Wohlfahrtiimonas chitiniclastica* (*W. chitiniclastica*) bloodstream infection associated with maggot therapy for a left temporal chronic wound.

## Case presentation

A 70-year-old male with a limited past medical history that was positive for stage I large B cell non-Hodgkin lymphoma diagnosed in 2012 but currently in remission after three cycles of chemotherapy presented to Sanford Medical Center Fargo for further evaluation and management of large left-sided temporal mass with evidence of secondary infection.

**Figure 1:**
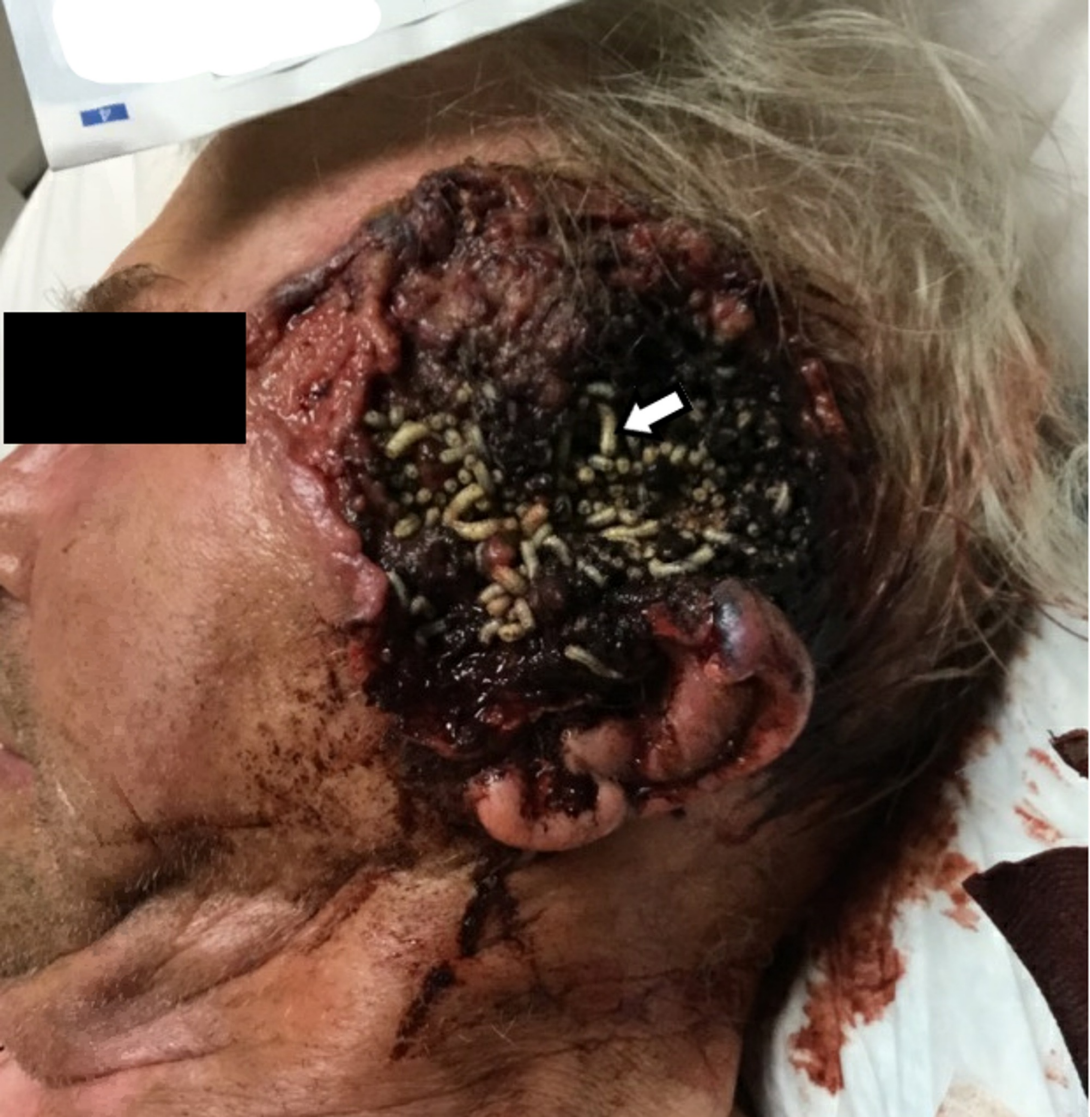
Elderly male presenting with a chronic left temporal wound managed with maggot therapy that resulted in Wohlfahrtiimonas chitiniclastica blood infection.

For over a year, he began to notice an ulcerating mass in the left temple, which slowly grew in size. He sought care from a naturopathic provider and maggot therapy was attempted. While on this treatment course, he had a mechanical fall and sought care in the emergency department. His vital signs were stable and was never febrile. He was noted to have a very large ulcerative and infiltrative soft tissue lesion in the left temple with evidence of secondary bacterial infection with associated maggot infestation (Figure 1). 

He was subsequently admitted for further management. White blood cell count was normal at 8.7 K/ul (reference range, 4-11) but inflammatory markers erythrocyte sedimentation rate and C-reactive protein were significantly elevated at 78 mm/hr (reference range, 0-15) and 107 mg/L (reference range, 0-8), respectively. Renal function was normal. He lives with his wife. He is retired and used to work on a variety of jobs including grounds keeping of a local school. He is a prior smoker and only drinks occasionally.

Otorhinolaryngology was consulted and performed tissue biopsy which revealed invasive squamous cell carcinoma. During his hospital course, he was noted to have a secondary bacterial infection of his left temple wound. Local cultures of the wound grew *Staphylococcus aureus* and *Proteus mirabilis*. Blood cultures grew W. chitiniclastica susceptible to quinolones, trimethoprim/sulfamethoxazole, cefepime, and piperacillin/tazobactam. He was able to be discharged after a week of hospitalization on oral levofloxacin. He eventually underwent extensive surgery including left total parotidectomy, facial nerve dissection, excision of the left cheek, scalp, including total auriculectomy, neck dissection, and left latissimus free tissue transfer with microvascular anastomosis followed by adjuvant radiation therapy which resulted in complete healing of the wound.

## Discussion

We present a case of *W. chitiniclastica* bacteremia associated with medical maggot therapy. It should be recognized that although maggot therapy has possible beneficial applications for debriding wounds, subsequent infection is a potential risk of treatment [1]. Maggots have been demonstrated to secrete defensins which are proteins similar to those produced by circulating human white blood cells that may result in the potential antimicrobial properties of maggot therapy [3]. However, an enigma exists regarding the presence and pathogenicity of the bacteria that maggots may inherently be associated with.

*W. chitiniclastica* is an aerobic, nonfermenting, oxidase-positive, gram-negative bacillus first isolated in 2008 from 3rd instar larvae of the fly *Wohlfahrtia magnifica*. The fleshfly, *W. magnifica* is an important parasitic fly that often affects livestock and occasionally humans in southern Europe, Asia, North Africa, and the Middle East [4]. In the United States, the common green bottle fly *Lucilia sericata* is a common cause of myiasis and has been implicated in one case of human *W. chitiniclastica* infection [5]. Transmission and potential subsequent infection of *W. chitiniclastica* are believed to be through the fly larvae in traumatic skin lesions or mucosal surfaces of the host [6].

Previous human cases of *W. chitiniclastica* have occurred globally including France, Argentina, Estonia, the United Kingdom, Japan, Australia, India, South Africa, Spain, and Malaysia, Hawaii, Germany, and the continental United States [7-17]. Infection is associated with social and medical circumstances that lead to poor sanitation, underlying medical conditions compromising normal host immune function, and chronic open wounds. Poor personal hygiene, lack of wound care, neurological disease or unconsciousness, alcoholism, and tobacco use were frequently described in clinical cases [5]. Patients often presented with wound infections, cellulitis, osteomyelitis, bacteremia, or sepsis [6].

Including this case, there were 14 patients in the literature with *W. chitiniclastica* bacteremia. Of these, five had a single pathogen in the blood. Three were primarily treated with a cephalosporin [7,15,17] while two cases including this one had a quinolone in the regimen. There were nine patient reports that had *W. chitiniclastica *as part of a polymicrobial blood infection and most utilized a beta-lactam antibiotic [5,6,9,11-13,18-20].

## Conclusions

*W. chitiniclastica *are rare causes of infections including bloodstream infections in humans. This case highlights the risk for *W. chitiniclastica* infection associated with maggots either through naturally occurring myiasis or with medical wound therapy of chronic wounds. Maggot therapy, though not routinely used, should only be practiced by qualified medical professionals who are able to weigh the risks and benefits.
